# The Bioactive Substance Secreted by MSC Retards Mouse Aortic Vascular Smooth Muscle Cells Calcification

**DOI:** 10.1155/2018/6053567

**Published:** 2018-06-03

**Authors:** Shuangshuang Wang, Maoqing Tong, Siwang Hu, Xiaomin Chen

**Affiliations:** ^1^Department of Cardiology, Ningbo Hospital of Zhejiang University, Ningbo 315000, China; ^2^Central Laboratory, Ningbo Hospital of Zhejiang University, Ningbo 315010, China; ^3^Spine Tumor Center, Changzheng Hospital, Second Military Medical University, Shanghai 200003, China

## Abstract

**Background:**

Vascular calcification, which is associated with low-level chronic inflammation, is a complication that occurs during aging, atherosclerosis, chronic kidney disease, diabetes mellitus, and hyperlipaemia. In this study, we used conditioned media from mesenchymal stem cells (MSC-CM), a source of autologous cytokines, to test the hypothesis that MSC-CM inhibits vascular smooth muscle cell (VSMC) calcification by suppressing inflammation and apoptosis.

**Methods:**

VSMCs were treated with *β*-glycerophosphate (*β*-GP) to induce calcification and MSC-CM was used as a treatment. Calcium deposition was evaluated using alizarin red and von Kossa staining after a 7-day induction period. Intracellular calcium contents were measured via the o-cresolphthalein complexone method, and alkaline phosphatase (ALP) activity was determined using the para-nitrophenyl phosphate method. The expressions of specific-osteogenic markers, inflammatory cytokines, and apoptosis-associated genes/proteins were examined by real-time polymerase chain reaction or western blotting.

**Results:**

MSC-CM inhibited *β*-GP-induced calcium deposition in VSMCs and decreased intracellular calcium content and ALP activity. Additionally, MSC-CM suppressed the *β*-GP-induced increases in BMP2, Msx2, Runx2, and osteocalcin expression. Additionally, MSC-CM decreased the expression of TNF-*α*, IL-1*β*, and IL-6 in VSMC. MSC-CM also partly blocked *β*-GP-induced VSMC apoptosis, which was associated with an increase in the Bcl-2/Bax expression ratio and a decrease in caspase-3 expression.

**Conclusion:**

Our study results suggest that MSC-CM can inhibit VSMC calcification. This suggests a potential novel clinical application for MSCs in the treatment of vascular calcification and associated diseases.

## 1. Introduction

Vascular calcification (VC) is a known complication of aging, coronary artery disease (CAD), hypertension, diabetes, and chronic kidney disease (CKD) and is strongly associated with cardiovascular mortality [1, 2]. Patients with diabetes, CAD, and CKD who exhibit extensive medial and intimal calcification have a significantly higher risk of cardiovascular mortality, compared with the general population. Given the dramatic increase in the global incidence of these diseases, an increase in the frequency of VC is expected in the next decade. Unfortunately, however, no ideal medical or other treatment options are currently available to prevent or treat this ongoing clinical problem.

VC research is often based on a cell model involving the differentiation of aortic vascular smooth muscle cells (VSMC) to osteocytes/chondrocytes, a process that plays an essential role in the initiation and progression of VC [1]. Currently, the close association of VC with inflammation is well known, and inflammatory cytokines have been shown to be crucial promoters of VSMC differentiation and VC [3, 4]. Additionally, increasing evidence suggests that apoptosis may be a critical mechanism underlying VC, as this process occurs prior to the initiation of VSMC calcification [5].

In the field of clinical medicine, various studies have identified the significant potential of mesenchymal stem cells (MSC) as effective therapeutic agents in various diseases such as peritonitis, sepsis, and myocardial infarction. The curative effects of MSCs in these disease settings may be partly attributed to the ability of these cells to secrete bioactive factors, including various cytokines as well as anti-inflammatory, antiapoptotic, and growth factors [6, 7]. Notably, conditioned media from MSCs (MSC-CM) contains autologous bioactive factors and is widely used in current clinical practice. We hypothesised that these potentially anti-inflammatory and antiapoptotic bioactive substances secreted by MSCs could prevent or treat VC. Herein, we describe the use of MSC-CM as a source of bioactive substances secreted by MSC and investigate its effects on VSMC calcification, as well as the underlying mechanism.

## 2. Materials and Methods

### 2.1. Cell Culture

Mouse MSCs were purchased from Cyagen Biosciences (Guangzhou, China), and mouse aortic VSMCs were purchased from Procell Life Science & Technology (Wuhan, China). In this study, we used MSCs between passages 3 and 6 and VSMCs between passages 8 and 11. VSMCs were routinely cultured in Dulbecco's Modified Eagle's Medium/High Glucose (H-DMEM) containing 4500 mg/L D-glucose and supplemented with 10% foetal bovine serum (FBS), 100 U/ml of penicillin, and 0.1 mg/ml of streptomycin. The cells were cultured in an incubator at 37°C in an atmosphere of 5% CO_2_ and were passaged using 0.5% trypsin/EDTA upon reaching confluence.

MSCs were cultured using the same complete medium. To maintain a consistent MSC-CM quality and ensure enough nutrient element left in MSC-CM, 2 × 10^6^ MSC were cultured in 15 ml H-DMEM for 48 hours. Subsequently, the MSC-CM was collected and centrifuged at 800 ×g for 10 minutes to remove detached MSCs and debris.

During experiments, VSMCs were treated with normal medium (control group), calcification medium containing 10 mM *β*-glycerophosphate (*β*-GP; Sigma, St. Louis, MO, USA) (H-DMEM^*β*-GP^ group), or MSC-CM containing 10 mM *β*-GP (MSC-CM^*β*-GP^ group). To ensure enough nutrients in the medium, 2 × 10^5^ VSMCs were cultured in 3 ml medium and the medium was changed every 2 days.

### 2.2. Alizarin Red Staining and Quantification

Mineral depositions in the experimental groups of VSMC were evaluated after a 7-day induction period. Calcified VSMCs were fixed with 4% paraformaldehyde for 1 hour at 4°C, followed by staining with 2% alizarin red (pH 4.2, Sigma) for 5 minutes at ambient temperature, two rinses in phosphate-buffered saline (PBS) to eliminate nonspecific staining, drying in an oven, and observation and imaging under an inverted microscope. To quantify alizarin red staining, 10% formic acid was used to elute the alizarin red dye taken up by VSMCs, and the absorbance of the resulting solution at 405 nm was detected and normalised to the protein content.

### 2.3. Von Kossa Staining

After a 7-day induction period, calcified VSMCs were detected using a von Kossa stain kit (Genmed Scientifics Inc., Wilmington, DE, USA). Cells were fixed in a 4% paraformaldehyde solution for 1 hour at 4°C, rinsed twice with ultrapure water, stained with 5% silver nitrate, and exposed to a 100-W light for 1 hour.

### 2.4. Intracellular Calcium Content

After a 7-day treatment with *β*-GP with or without MSC-CM, VSMCs were washed twice with PBS and decalcified in 0.6 mM HCl at 4°C for 24 hours. The calcium content in the supernatant was detected using a Calcium Colorimetric Assay kit (BioVision, Milpitas, CA, USA).

### 2.5. Alkaline Phosphatase (ALP) Activity Assay

The ALP activity in VSMCs was measured using LabAssay ALP (Wako Pure Chemical Industries, Osaka, Japan). Briefly, VSMCs were treated in medium supplemented with or without *β*-GP for 7 days. Subsequently, the cells were washed and subjected to three freeze–thaw cycles in 0.1% Triton X-100 solution. Twenty microlitre aliquots of the resultant cell lysates were mixed with 100 *μ*l of p-NPP substrate and incubated at 37°C for 15 minutes, after which the absorbances of the solutions at 405 nm were measured using a microplate reader. The ALP activity was normalised to the protein content of each sample, which was determined using a bicinchoninic acid protein assay.

### 2.6. Cell Viability

A cell counting kit-8 (CCK-8, Dojindo Laboratories, Kumamoto, Japan) was used to evaluate cell viability. VSMCs (5 × 10^3^ cells/well) were seeded into 96-well plates and incubated at 37°C for various time points (1, 2, 3, 4, and 5 days). At each time point, the cell medium in each well was replaced with 100 *μ*l of fresh complete medium and 10 *μ*l of CCK-8. After a 1-hour incubation with CCK-8 at 37°C, the absorbance at 450 nm was measured.

### 2.7. Flow Cytometric and Hoechst Fluorescence Analysis of Apoptosis

An Annexin V-FITC/Propidium Iodide (PI) Apoptosis Detection Kit (Dojindo Laboratories, Kumamoto, Japan) was used for the VSMC apoptosis assay. After a 3-day treatment period, VSMCs were removed from culture plates via trypsin digestion and washed twice with PBS. Following resuspension, the cells were double-stained with annexin V-FITC and PI and analysed via flow cytometry (FACSCanto, BD Biosciences, San Jose, CA, USA). After gating, the apoptosis rate was determined as the sum of the cell populations in quadrant 3 (Q3) (FITC+/PI−) and Q2 (FITC+/PI+). For Hoechst staining, VSMCs were treated for 3 days and subsequently stained with Hoechst 33258 (Merck, Kenilworth, NJ, USA) for 10 min in the dark. Morphological changes such as chromosomal condensation and nuclear fragmentation were counted in five different fields under a fluorescence microscope (magnification ×200).

### 2.8. Quantitative Real-Time PCR Analysis

Total RNA was extracted from cultured VSMCs using TRIzol Reagent (Invitrogen Corp, Carlsbad, CA, USA). Subsequently, 2 *μ*g of total RNA were reversed-transcribed to cDNA using a RT-PCR kit (TaKaRa Biotech, Shiga, Japan). The levels of mRNA corresponding to BMP2, Msx2, Runx2, and osteocalcin were quantitated in cells treated for 3 days, whereas those corresponding to TNF-*α*, IL-1*β*, IL-6, caspase-3, Bax, and Bcl-2 were quantitated after a 1-2-day treatment period. RT-PCR was performed using an ABI 7500HT Fast Real-Time PCR System (Applied Biosystems, Foster City, CA, USA). GAPDH was used as an endogenous control.

### 2.9. Western Blotting

Cells were harvested and homogenised after 3–7 days of treatment. The cell homogenates were subjected to SDS–polyacrylamide gel electrophoresis and protein transfer, and the resulting PVDF membranes were incubated with appropriate antibodies specific for BMP-2, Runx2, TNF-*α*, caspase-3, and *β*-actin (all from Abcam, Cambridge, UK). The reactions were visualised using enhanced chemiluminescence.

### 2.10. Statistical Analysis

Data are presented as means ± standard deviations. Differences among multiple groups were assessed using a one-way analysis of variance (ANOVA). A *P* value < 0.05 was considered statistically significant.

## 3. Results

### 3.1. MSC-CM Suppresses Mineral Deposition and ALP Activity in VSMCs

**Figure 1:**
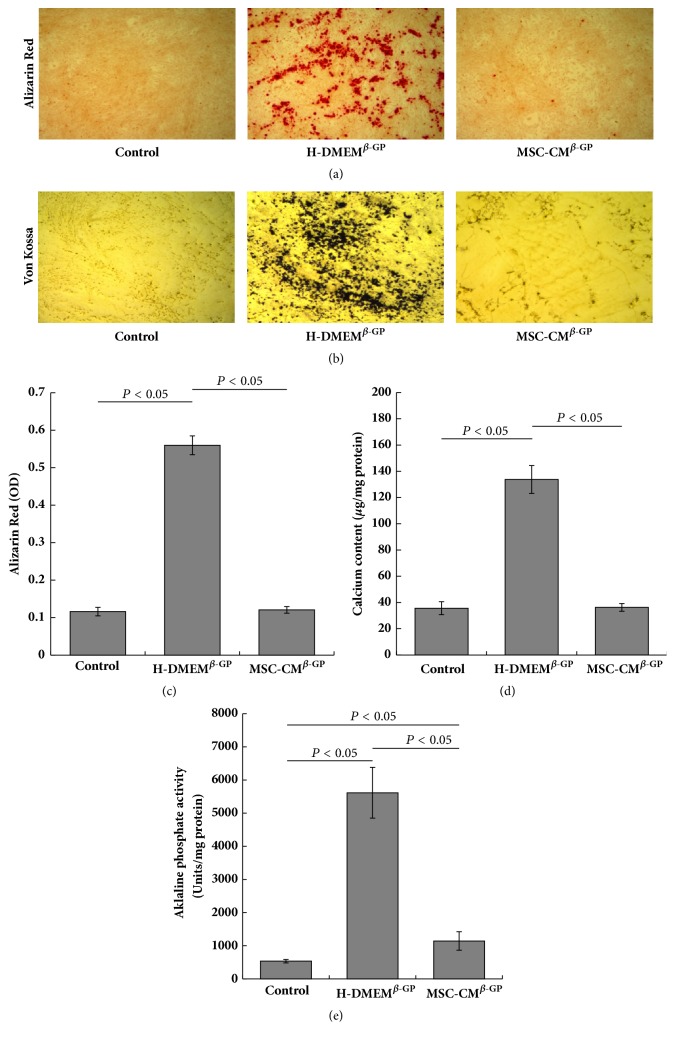
MSC-CM suppresses mineral deposition and ALP activity in VSMCs. VSMCs from different treatment groups were incubated for 7 days. Images of (a) alizarin red staining (magnification ×100) and (b) von Kossa staining (×100). The graphs depict the quantification of alizarin red staining (c), calcium content (d), and ALP activity (e) analyses.

To investigate the effects of MSC-CM effect on VSMC calcification, we subjected cells exposed to different types of media for 7 days to alizarin red staining and quantification, as well as von Kossa staining. Almost no mineral deposits were observed in the control cells, whereas VSMCs treated with 10 mM *β*-GP (H-DMEM^*β*-GP^ group) had a relatively high level of mineral deposition (Figures 1(a)–1(c)). Compared with the H-DMEM^*β*-GP^ group, VSMCs in the MSC-CM^*β*-GP^ group contained fewer mineral deposits, which suggests that MSC-CM inhibited *β*-GP-induced mineralisation in VSMCs.

To further investigate whether MSC-CM could efficiently prevent the differentiation of VSMCs to osteoblasts, we measured the intracellular calcium content and ALP activity. The H-DMEM^*β*-GP^ group had a significantly higher calcium content relative to the control group (Figure 1(d)). However, the high calcium level induced by *β*-GP was dramatically reduced by treatment with MSC-CM (MSC-CM^*β*-GP^ group). Similarly, a remarkable increase in ALP activity was observed in *β*-GP-treated VSMCs (H-DMEM^*β*-GP^ group) relative to the control group. Again, this increase was significantly decreased by MSC-CM (MSC-CM^*β*-GP^ group) (Figure 1(e)). These data demonstrate the ability of MSC-CM to attenuate the osteoblastic differentiation of VSMCs and indicate that MSC-CM might be a novel therapeutic option for blocking VSMC calcification.

### 3.2. MSC-CM Inhibits the Expression of Specific-Osteogenic Markers in VSMCs

**Figure 2:**
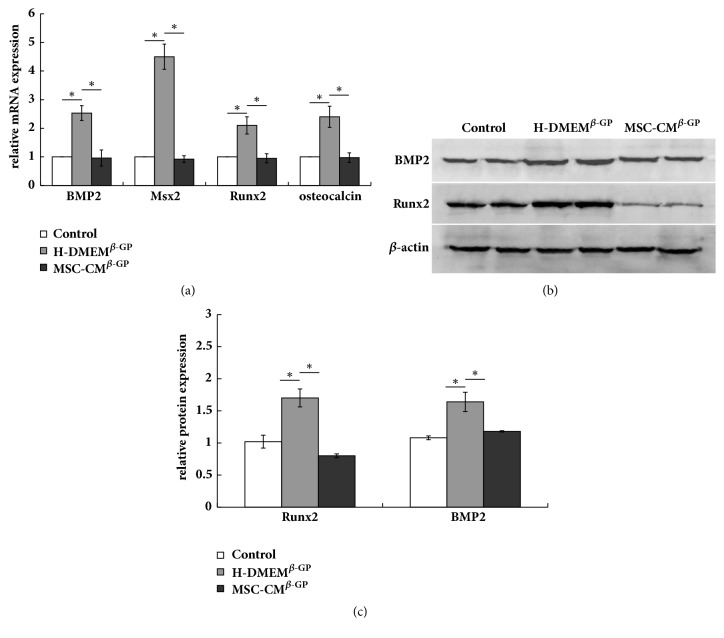
MSC-CM inhibits the expression of osteogenesis-specific markers in VSMCs. (a) The mRNA expression levels of BMP-2, Msx2, Runx2, and osteocalcin were determined by RT-PCR after 3 days. (b) The protein expression levels of BMP-2 and Runx2 were measured by western blotting; *β*-actin was used as an endogenous control. (c) BMP-2 and Runx2 protein levels were quantified densitometrically. ^*∗*^*P* < 0.05.

To further verify the suppressive effects of MSC-CM on VSMC calcification, we evaluated the mRNA expression of osteogenesis-specific markers, such as BMP2, Msx2, Runx2, and osteocalcin, in VSMCs after a 3-day induction period. The relative mRNA expression levels of all four markers were increased in the H-DMEM^*β*-GP^ group relative to the control group. However, these *β*-GP-induced increases in marker mRNA expression were abolished by treatment with MSC-CM (MSC-CM^*β*-GP^ group) (Figure 2(a)). Furthermore, we detected the protein expression of BMP2 and Runx2, which are more classic and important indicators in vascular calcification. Similarly, the protein expression levels of BMP2 and Runx2 were upregulated in the H-DMEM^*β*-GP^ group, compared with the control group, whereas this upregulation was significantly suppressed by treatment with MSC-CM (MSC-CM^*β*-GP^ group) (Figures 2(b) and 2(c)).

### 3.3. MSC-CM Suppresses *β*-GP-Induced Inflammatory Cytokine Expression in VSMCs

**Figure 3:**
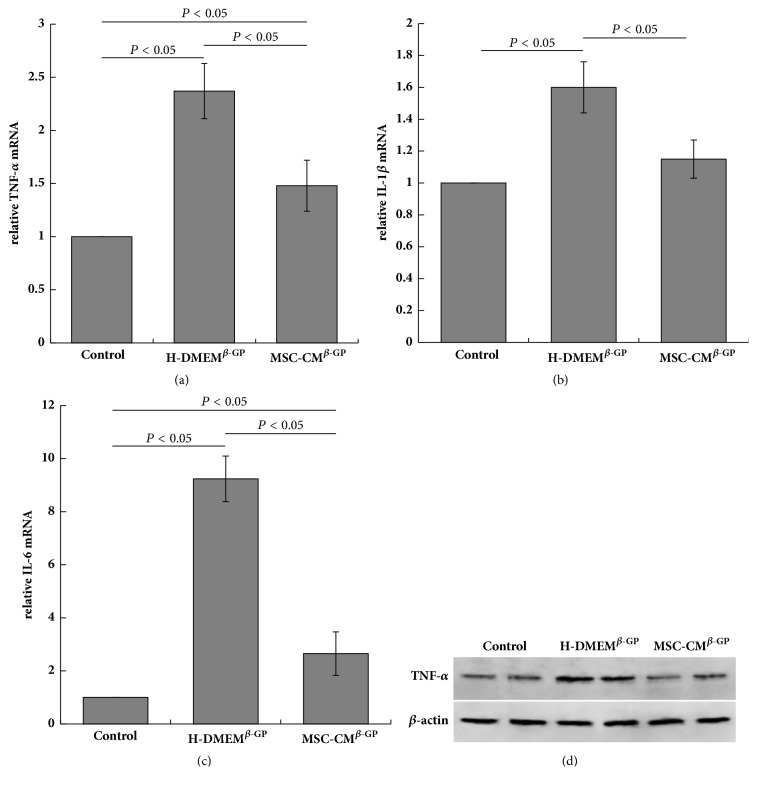
MSC-CM suppresses *β*-GP-induced inflammatory cytokine expression in VSMCs. (a–c) Expression levels of TNF-*α*, IL-1*β*, and IL-6 mRNA are shown. (d) TNF-*α* protein expression was determined by western blotting; *β*-actin was used as an endogenous control.

TNF-*α*, IL-1*β*, and IL-6 are important inflammatory cytokines with crucial roles in the initiation and progression of vascular calcification. To further examine the mechanisms by which MSC-CM inhibits VSMC calcification, the levels of inflammatory cytokines expressed by VSMC were determined using RT-PCR and western blotting. Notably, VSMCs exposed to *β*-GP (H-DMEM^*β*-GP^ group) expressed higher levels of TNF-*α*, IL-1*β*, and IL-6 mRNA, compared with untreated control cells (control group). These *β*-GP-induced increases in inflammatory cytokine expression were suppressed by MSC-CM (MSC-CM^*β*-GP^ group) (Figures 3(a)–3(c)). Furthermore, TNF-*α* protein levels were upregulated after *β*-GP treatment, and this phenomenon was reversed by the addition of MSC-CM as shown in Figure 3(d).

### 3.4. MSC-CM Suppresses the *β*-GP-Induced Apoptosis of VSMCs

**Figure 4:**
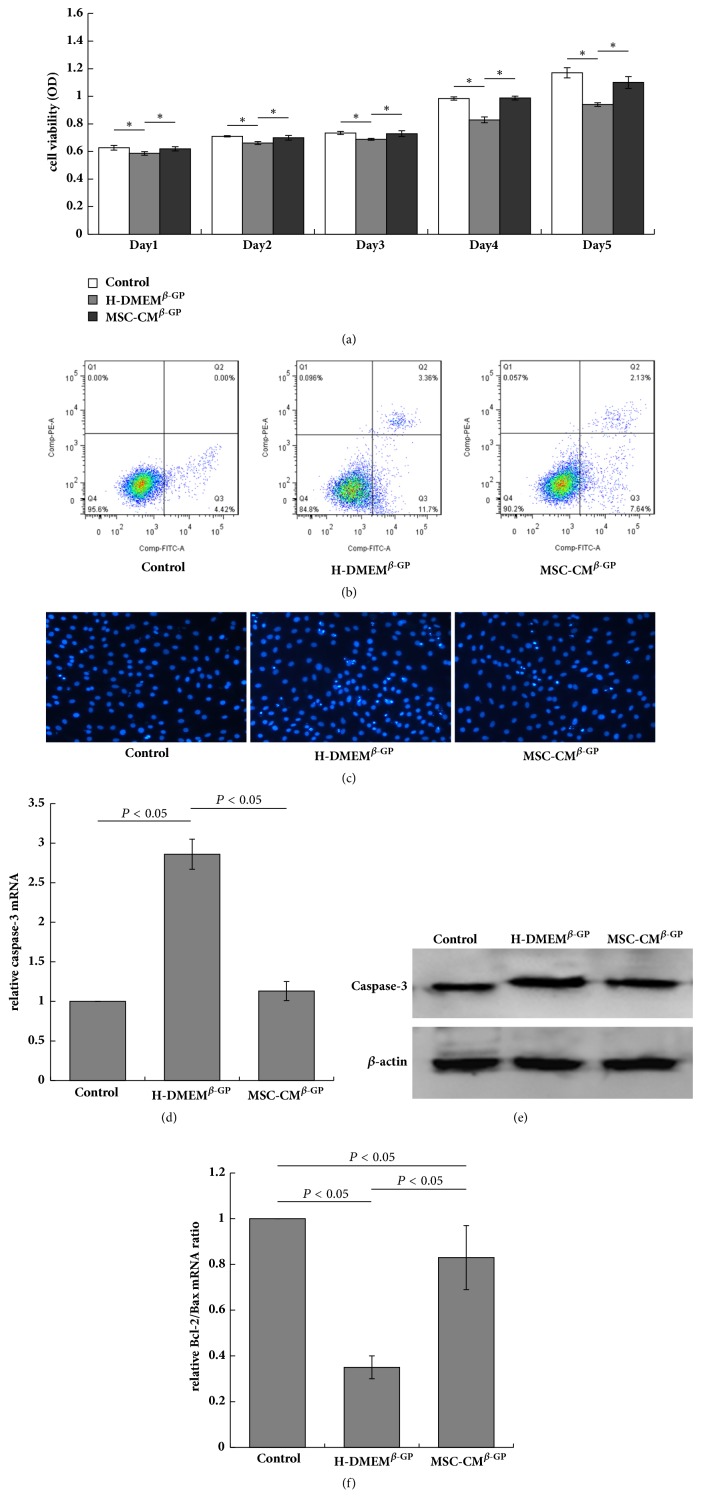
MSC-CM prevents *β*-GP-induced VSMC apoptosis. (a) A CCK8 assay was used to test VSMC viability (^*∗*^*P* < 0.05). (b) The frequency of VMSC apoptosis was analysed using flow cytometry and calculated as quartile 2 (Q2) (FITC+/PI+) + Q3 (FITC+/PI−). (c) Apoptotic cells were determined among Hoechst-stained VSMCs (×200). (d) The relative mRNA expression (e) and protein expression of caspase-3 were determined by RT-PCR and western blotting, respectively. (f) The Bcl-2/Bax mRNA expression ratio was determined by RT-PCR. ^*∗*^*P* < 0.05.

Many studies have identified *β*-GP as an inducer of apoptosis in VSMCs through a process involving the release of inorganic phosphate and consequent VSMC calcification. These earlier findings suggest that apoptosis plays a causative role in VSMC calcification [8]. To validate the mechanisms by which MSC-CM suppresses VSMC calcification, we measured viability and apoptosis in the experimental cell groups. First, we used a CCK8 assay to measure the viability of VSMCs exposed to different culture conditions. Notably, cells in the H-DMEM^*β*-GP^ group exhibited reduced viability relative to those in the control group at every time point, whereas cells in the MSC-CM^*β*-GP^ group exhibited increased cell viability relative to the H-DMEM^*β*-GP^ group (Figure 4(a)).

We next analysed VSMC apoptosis using flow cytometry. In the H-DMEM^*β*-GP^ group, exposure to *β*-GP strongly increased the rate of apoptosis among VMSCs relative to the control group (14.57 ± 1.17% versus 4.26 ± 0.46%). However, this *β*-GP-mediated increase in apoptosis was remarkably reversed by MSC-CM (9.71 ± 0.54% in the MSC-CM^*β*-GP^ group) (Figure 4(b)). Hoechst staining revealed the same nuclear changes in all three groups (Figure 4(c)).

Cell apoptosis is regulated by the expression of caspase-3 and the ratio of the antiapoptotic factor Bcl-2 to the proapoptotic factor Bax [9]. Therefore, we evaluated the expression of caspase-3 and ratio of Bcl-2/Bax in VSMCs using RT-PCR and western blot as a further measure of the effects of MSC-CM. Compared to the control group, both the mRNA and protein levels of caspase-3 were significantly increased in VSMCs from the H-DMEM^*β*-GP^ group, whereas these increases were inhibited by MSC-CM treatment (MSC-CM^*β*-GP^ group) (Figures 4(d) and 4(e)). Additionally, the Bcl-2/Bax mRNA expression ratio was reduced in the H-DMEM^*β*-GP^ group relative to the control group; again, this effect was suppressed by MSC-CM (MSC-CM^*β*-GP^ group) (Figure 4(f)). These results suggest that MSC-CM exerts antiapoptotic effects on *β*-GP induced VSMC apoptosis. This effect may be closely associated with the ability of MSC-CM to inhibit calcification in VSMCs.

## 4. Discussion

Currently, the prevalence of vascular calcification (VC) is increasing in accordance with growth in the populations of elderly and dysmetabolic adults [10, 11]. VC, in which calcium deposits in the coronary arteries affect atherosclerotic plaque stability and increase the incidence of the acute coronary syndrome, is frequently observed in aging and various diseases, including atherosclerosis, diabetes, chronic kidney disease, hypertension, osteoporosis, and hyperlipaemia [12, 13]. Accumulating evidence has identified inflammation and apoptosis as two potentially important mechanisms underlying the initiation and progression of VC and thus provides possible clues regarding the prevention and control of this condition [14].

MSC-based medical therapies have emerged as potential cell-based treatment options for many disorders, including brain injury [15], lung diseases [16], myocardial infarction [17], spinal cord injury [18], and renal injury [19]. The potential benefits of these cells are attributed mainly or partly to anti-inflammatory and antiapoptotic properties. In our study, we identified a powerful anticalcification effect of MSC-CM, which almost completely inhibited *β*-GP-induced mineral deposition (e.g., alizarin red and von Kossa staining). In addition, MSC-CM also dramatically inhibited the *β*-GP-induced increase in intracellular calcium levels and reduced the activity of ALP, an important enzyme in early osteogenesis. Furthermore, MSC-CM abolished the upregulation of various markers of calcification and osteogenesis, including BMP2, Msx2, Runx2, and osteocalcin, and mitigated *β*-GP-induced increases in inflammation and apoptosis in VSMCs. Taken together, these results indicate that MSC-CM can inhibit VSMC calcification and therefore may be a new therapeutic option for the prevention or control of VC.

Increasingly, studies have reported that VSMCs undergo a shift to an osteoblastic phenotype during the onset and progression of VC, and this change is accompanied by the upregulation of specific markers of osteogenesis (e.g., BMP-2, Runx2, Msx2, and osteocalcin) [20–22]. Accordingly, the levels of these markers correlate with the level of VC. Zhang et al. reported that IL-18 significantly promotes VC, as well as increasing in the expression of BMP-2, Runx2, and osteocalcin, in VSMC [23], while Alesutan demonstrated that homoarginine augmented VC and increased mineral deposition, ALP activity, and Msx2 mRNA expression in human aortic smooth muscle cells [24]. Accordingly, we evaluated the expression of these osteogenic markers in VSMCs to further investigate the inhibitory effect of MSC-CM on calcification and found that *β*-GP led to an increase in calcification and upregulation of these osteogenic markers, consistent with previous studies. By contrast, MSC-CM abolished these effects of *β*-GP, thus suggesting that MSC-CM warrants further consideration as a potential therapeutic option for the prevention or alleviation of VC.

MSCs mainly or partly exert their effects via the production of autocrine or paracrine bioactive substances, such as anti-inflammatory, antiapoptotic, and growth factors [25]. Notably, inflammation is a key contributor to the development and progression of vascular calcium deposition [26, 27], which has itself been identified as an active inflammatory process. Furthermore, some level of inflammation at different vascular sites, including the intima, media, and even adventitia, has been identified as a very common phenomenon in most forms of arterial calcification [28–30]. Previous studies have confirmed the involvement of various cytokines in VC: for example, TNF-*α* accelerates calcium deposition in VSMC by provoking the release and strengthening the activity of BMP-2 [4]. Furthermore, IL-1*β* stimulates tissue-nonspecific ALP activity and calcification in VSMCs [30], while IL-6 enhances the activity of BMP-2 by inhibiting matrix gla protein (MGP) [31]. Consistent with many previous studies [32, 33], we also demonstrated that exposure to *β*-GP stimulates inflammatory reactions in VSMCs, as indicated by the enhanced expression of various inflammatory cytokines, including TNF-*α*, IL-1*β*, and IL-6. Moreover, we found that the levels of these three cytokines decreased significantly in MSC-CM-treated VSMCs relative to their *β*-GP-treated counterparts. Similarly, Chen et al. reported that MSC-CM significantly decreased the expression of TNF-*α*, IL-1*β*, and IL-6 mRNA in irradiated rat livers [34]. These findings suggest that MSC-CM protects against *β*-GP induced inflammation, which may be a key mechanism underlying the ability of MSC-CM to inhibit VSMC calcification. Although experts believe that MSCs exert their anti-inflammatory effects via the production and release of bioactive substances such as tumour necrosis factor alpha-stimulated gene-6, prostaglandin E2, indoleamine 2,3-dioxygenase, inducible nitric oxide synthase, and IL-10 [7], the exact bioactive substances secreted by MSCs and the related molecular mechanisms will require in-depth research in the future.

VSMC apoptosis has been reported to accompany calcification induced by exposure to high phosphate levels [35, 36], and the association between apoptosis calcification was verified by the observation that the inhibition of caspase-mediated apoptosis also reduced calcification [5]. In VSMCs, apoptosis precedes calcification, and apoptotic bodies may act as nucleating structures for calcium deposition. Notably, both MSCs and MSC-CM have been shown to exert antiapoptotic effects in various diseases. Xagorari et al. found that MSC-CM activated FGL1 to protect against hepatic cell apoptosis following acute liver injury [37], and Huang et al. reported that paracrine factors secreted by MSCs could protect astrocytes from apoptosis [38]. To further explore the mechanisms by which MSC-CM suppresses VSMC calcification, we evaluated the effects of MSC-CM on VSMC viability, apoptosis, and the expression of associated markers. Consistent with a previous report [39], we verified that *β*-GP promotes VSMC apoptosis by reducing the ratio of Bcl-2/Bax expression and increasing the expression of caspase-3. By contrast, MSC-CM suppresses *β*-GP-induced apoptosis by increasing the ratio of Bcl-2/Bax expression and reducing the expression of caspase-3, suggesting that the inhibitory effects of MSC-CM on VSMC calcification are at least partly mediated via antiapoptotic mechanisms.

Although our study revealed that MSC-CM might inhibit VSMC calcification via its anti-inflammatory and antiapoptosis properties, we did not determine which bioactive substance secreted by MSCs is responsible for these effects. The exact molecular mechanism responsible for this cytoprotective effect can only be defined by purifying and identifying this putative bioactive substance. However, this process is a complex, long-term project that is beyond the scope of this article.

In conclusion, our findings are the first to implicate MSC-CM as a protector against calcification in vitro via reductions in inflammation and apoptosis, which play pivotal roles in vascular calcification. Therefore, we have identified a novel potential therapeutic agent for VC and associated diseases, such as atherosclerosis, diabetes, and chronic kidney disease. Nevertheless, we are not sure which bioactive substance secreted or consumed by MSCs is responsible for these effects. In our study, we supplied MSCs with enough H-DMEM to avoid serum or other medium content deprivation. Thus, we tend to believe that the bioactive substance secreted by MSCs works in these effects. However, identification of the bioactive substance present in MSC-CM that exerts these protective effects will require further studies.

## Data Availability

All the data are available from Dr. Shuangshuang Wang (wangss1023@126.com) upon request.
